# Effects of Replacing Dietary Fishmeal with Enzyme-Treated Soybean Protein on Growth Performance, Serum Biochemical Indices, Antioxidant, Digestive Enzyme Activities, Intestinal Barrier Integrity and Relative mRNA Expression Levels of Juvenile Largemouth Bass (*Micropterus salmoides*)

**DOI:** 10.3390/ani16162589

**Published:** 2026-08-19

**Authors:** Xia Dong, Jie Guo, Hongsen Xu, Pengcheng Xiong, Luyang Li, Lihe Liu, Yulan Liu, Xiangyu Wang, Xiaolong Li, Honggang Huang, Xiao Xu

**Affiliations:** 1 Hubei Key Laboratory of Animal Nutrition and Feed Science, School of Animal Science and Nutritional Engineering, Wuhan Polytechnic University, No. 68, Xuefunan Road, Wuhan 430023, China; dx_whpu2024@163.com (X.D.); hongsenxu@whpu.edu.cn (H.X.); 18672900509@163.com (P.X.); 17371453782@163.com (L.L.); liulihe06@126.com (L.L.); yulanflower@126.com (Y.L.); 2 Nutrition & Health Research Institute, COFCO Corporation, Beijing 102209, China; guojie6@cofco.com (J.G.); wang_xiangyu@cofco.com (X.W.); li-xiaolong@cofco.com (X.L.)

**Keywords:** largemouth bass, enzyme-treated soy protein, growth, immunity, intestinal barrier function

## Abstract

The enzyme-treated soybean protein (ETSP) could improve their application as an ingredient and substitute for fishmeal (FM) in aquafeeds. In the present research, an eight-week feeding trial was carried out to investigate the effects of using ETSP as a particle substitution in FM on growth performance, serum biochemical indices, antioxidant, intestinal barrier integrity and relative mRNA expression levels in largemouth bass (*Micropterus salmoides*). A cohort of 450 fish (15.52 ± 0.04 g/fish) were randomly divided into five groups with five replacement levels of ETSP: 0, 12.5, 25.0, 37.5 and 50.0% (control, H2-12.5, H2-25.0, H2-37.5 and H2-50.0). Results showed that final body weight (FBW), weight gain (WG), and specific growth rate (SGR) reach the highest value, and the feed conversion ratio (FCR) reaches lowest in H2-25.0 group. The activities of total superoxide dismutase (T-SOD), catalase (CAT) and glutathione peroxidase (GSH-Px) were remarkably increased by replacing FM with ETSP at certain level, while malondialdehyde (MDA) concentration significantly decreased (*p* < 0.05). Higher levels of total protein (TP) and albumin (ALB) as well as lower activity of aspartate aminotransferase (AST) were detected with ETSP replacement. Histological examination revealed that villus height in mid-intestine was the highest, while this factor in pyloric caeca was not affected. The activity of diamine oxidase (DAO) and the contents of D-lactate (D-Lac) and lipopolysaccharide (LPS) in serum were gradually decreased when replacing FM with ETSP in the diets. In addition, replacement of FM with ETSP at 25% improved intestinal barrier function by upregulating gene expression related to the tight junctions (*occludin*, *claudins*, *ZO-1* and *ZO-2*) and anti-inflammatory cytokines (*IL-10*) while downregulating pro-inflammatory factors (*IL-8* and *TNFα*). Moreover, proper amount of ETSP instead of FM can upregulate mRNA expression of *LAT1*, *PEPT1* and *PEPT2* in the intestine. In conclusion, at the fishmeal level used in the diet tested in the present study, 25% replacement is the most beneficial for juvenile largemouth bass.

## Simple Summary

Fishmeal (FM) is a traditional but expensive and limited feed ingredient, so finding sustainable alternatives is important for aquaculture. This study investigated whether enzyme-treated soybean protein (ETSP) could effectively replace fishmeal in the diet of farmed largemouth bass. In an eight-week feeding trial, we replaced 0%, 12.5%, 25%, 37.5%, and 50% of FM with ETSP in the fish diets. The results demonstrated that replacing 25% of FM with ETSP has the best growth performance, implying the fish grew faster and used feed more efficiently. Additionally, fish fed with this optimal level increased antioxidant activity and improved intestinal structure and barrier function. Moreover, replacement of FM with ETSP at 25% enhanced expression of genes related to nutrient transport, tight junction proteins, and anti-inflammatory responses. Overall, at the fishmeal level used in the diet tested in the present study, 25% replacement is the most beneficial for juvenile largemouth bass.

## 1. Introduction

In aquafeeds, fish meal (FM) is a highly desirable protein source because of the well-balanced amino acid composition, protein levels, high digestibility, ideal nutrient profile, unsaturated fatty acids, and so on [1], whereas the overexploitation of fisheries and the rising price of FM seriously restrict its use in aquatic feed [2]. Reducing dietary FM inclusion in the diets not only helps lower feed expenses but also meets current directions in aquafeed innovation. Hence, identification, development, and utilization of FM alternatives with suitable protein sources have been become a high-priority strategy to satisfy the sustainable aquaculture development [3].

Soybean meal, valued for its relatively low cost, wide availability, bioactive compounds and balanced amino acids and high protein content, is becoming the ideal plant-based alternative for partially replacing FM in aquafeeds [4]. Nevertheless, indigestible carbohydrates, antinutritional factors, and low essential amino acid levels partially limit the use of soybean as a major protein source for aquatic animals [5]. The over substitution of FM by soybean resulting in the reduced growth, poor feed efficiency, lower feed intake, and potentially increased mortality in fish [6]. Moreover, gut inflammation could be induced by high use of soybean in animals [7], especially in carnivorous fish species, such as largemouth bass (*Micropterus salmoides*) [8] and snakehead (*Channa argus*) [9].

The treatment of soybean proteins with different kinds of proteases is a promising method used to eliminate the antinutritional components under mild conditions while causing effective antinutritional factor removal and minimal nutrient loss [10]. Moreover, enzymatic hydrolysis converts soybean macromolecules (proteins, starch, cellulose, and mannans) into smaller, readily absorbable components including peptides, amino acids, glucose, and maltose [11]. Additionally, large fat molecules are also emulsified into smaller particles, making them easier for fish to digest. It has been well documented that enzymatically derived soybean peptides could improve digestive enzyme activities, thereby promoting the growth and development of fish larvae [12].

The largemouth bass (*M. salmoides*) is a commercially significant species owing to its fast growth rate, cold tolerance, and high nutritional and economic value [13]. Native to the Southeastern US, the largemouth bass was firstly introduced to China in the 1980s and has since become a major freshwater fish [14]. By 2024, China’s annual output in largemouth bass production had reached 938 thousand tons, making it the global leader [15]. As a carnivorous fish, the largemouth bass requires a diet containing 40–55% protein on a dry matter basis [16]. Even though some studies have investigated the effect of enzyme-treated soybean protein to replacing FM, the potential role in molecular mechanism, such as amino acid transports, peptide transports and tight junction components, was limited. This study investigates the effects of enzyme-treated soybean protein as am FM replacer on juvenile largemouth bass growth, nutritional metabolism, antioxidant status, non-specific immunity, digestive enzyme activity and gene expression.

## 2. Materials and Methods

### 2.1. Enzyme-Treated Soybean Protein

The enzyme-treated soybean protein (ETSP) with high small peptides was provided by China National Food and Oil Corporation (Beijing, China). In brief, the 48% dehulled soybean meal from oil plant was firstly subjected to solvent extraction at 50 °C. The solvent-extracted soybean meal was further hydrolyzed using in-house produced alkaline protease (750 IU/g), neutral protease (250 IU/g), acid protease (250 IU/g), α-Galactosidase (500 IU/g), mannanase (500 IU/g) and phytase (500 IU/g) at 50 °C and a pH of 8.0–9.0 for a duration of 12 h. Afterwards, the intermediate product was heated at 105 °C for 30 min to inactivate enzymes, followed by drying at 80 °C for 15 h. The compositions of proximate and amino acid in the ETSP were detected and shown in the Supplemented Tables S1 and S2.

### 2.2. Experimental Diets

In this study, four isonitrogenous (46.6% crude protein) and isolipidic (10.1% crude lipid) diets were formulated by replacing 0% (control group), 12.5% (H2-12.5), 25.0% (H2-25.0), 37.5% (H2-37.5) and 50.0% (H2-50.0) of FM proteins with ETSPs. Using α-cellulose as a filler, five experimental diets were supplemented with methionine and lysine to achieve a balance of essential amino acids. Table 1 shows the formulation and proximate composition of these diets. Dry ingredients were pre-weighed and mixed using a Hobart mixer (M-256, South China University of Technology, Guangzhou, China) for 15 min prior to pelleting. Boiling water (35% by weight) was then added to attain a consistency suitable for pelleting. The moist mash from each diet was subsequently pelleted into 1 mm^−3^ diameter pellets using pelletizer (Y90L-2, Xinchang Chenshi Machinery Co., Ltd., Ningbo, China). All diets were subjected to drying in a ventilated oven at 60 °C for roughly 12 h to achieve a moisture content of <10%, then kept frozen at −20 °C prior to use.

### 2.3. Experimental Fish and Feeding Management

The feeding experiment was carried out at the Wuhan Polytechnical University. Juvenile largemouth bass (*M. salmoides*) with average weight of 15.52 ± 0.04 g were sourced from a Bass Breeding Base in Wuhan, Hubei, China. Before the feeding experiment, the fish were acclimated in a recirculating aquaculture system (RAS) for two weeks. Following that, a total of 450 fish were randomly chosen, batch-weighed and assigned to 15 fiber glass tanks (300 L) at a density of 30 fish per tank. Each tank was provided with flow-through system with continuously aerated water at a rate of approximately 1 L per min, and the water temperature was maintained at 25.00–29.00 °C. All tanks were maintained under a natural light cycle. Throughout the feeding trial, the Ammonia-N, dissolved oxygen and pH were monitored daily and maintained at 0.02 ± 0.01 mg/L, 7.80 ± 0.30 mg/L and 7.50 ± 0.30, respectively. Fish were fed up to apparent satiation at 10:00 and 17:00 daily at rate of 2% body weight for eight weeks, with every three tanks randomly assigned to one of the experimental diet groups. Feces and uneaten feed were removed at one hour post feeding by siphon from the bottom of each tank.

### 2.4. Sample Collection

At the end of the experiment, all fish were deprived of feed for 24 h. Subsequently, the fish from each tank were anesthetized using MS-222 (Sigma, Ronkonkoma, NY, USA) at 0.1 g/L for weighting and counting growth and feed intake. From each group, six fish (two fish from each replicate tank) were randomly chosen and frozen at −20 °C for subsequent determination of whole-body proximate composition. The blood was taken from caudal veins of three fish per tank, placed into 1.5 mL tubes, incubated at room temperature for 1 h. Serum was separated by centrifugation (1200× *g*, 10 min, 4 °C) and preserved at −80 °C for future use. Tissue samples of mid-intestine and pyloric caeca were dissected from aforementioned blood-sampled fish and subsequently preserved in 4% polyoxymethylene to allow for histological analysis. Moreover, the intestines were sampled from another 9 fish and stored at −80 °C for future analyzing of digestive enzyme activities. For target gene expression analysis, intestinal samples were collected from another six fish per group, preserved in RNA later (TaKaRa, Dalian, China) at −80 °C, and then used for reverse-transcriptase polymerase chain reaction (qRT-PCR) analysis. The livers from the same fish were also sampled, rinsed with sterile PBS, transferred into 1.5 mL tubes and immediately stored at −80 °C for subsequent antioxidant capacity assessment.

### 2.5. Growth Performance, Feed Utilization and Physical Indices

After 8 weeks of feeding, all fish were fasted for 24 h, then counted and weighed to determine survival rate (SR), weight gain (WG), specific growth rate (SGR) and feed conversion ratio (FCR). Six fish per tank were randomly selected, weighed, and measured their body length to calculate condition factor (CF). Blood was drawn from the caudal vein, subsequently centrifuged at 4 °C for 10 min (1200× *g*) and stored at −80 °C for future study of immune enzyme activity. After the bleeding, the fish were dissected and viscera, liver, intestine, stomach and intestinal lipid were taken and weighed to determine viscera somatic index (VSI), liver somatic index (LSI), intestine somatic index (ISI) and stomach somatic index (SSI), intraperitoneal fat ratio (IFR), respectively.

The parameters were calculated by the formulas listed below:

SR (%) = 100 × final number of fish/initial number of fish

WG (g) = FBW (g) − IBW (g)

SGR (%/day) = 100 × (Ln FBW − Ln IBW)/T

FCR = feed intake (g)/WG (g)

CF (g/cm^3^) = 100 × body weight (g)/body length (cm)^3^

VSI (%) = 100 × viscera weight (g)/body weight (g)

LSI (%) = 100 × liver weight (g)/body weight (g)

ISI (%) = 100 × intestine weight (g) 100/body weight (g)

SSI (%) = 100 × stomach weight (g)/body weight (g)

IFR (%) = 100 × intraperitoneal fat weight (g)/body weight (g).

where IBW represents initial body weight, FBW is final body weight and T indicates the number of days in the feeding period.

### 2.6. Analysis of Diets and Fish Body Composition

The levels of moisture, crude protein, crude lipid and ash in both experimental diets and fish samples were measured according to the protocols of the Association of Official Analytical Chemist (AOAC) [17]. Moisture was obtained by drying at 105 °C to a constant weight, crude protein was calculated as nitrogen × 6.25, total crude lipid was determined following the ether extraction protocol of Folch et al. [18], and ash content was measured after burning samples at 550 °C for 6 h in a muffle furnace. Each sample was analyzed in duplicate.

### 2.7. Serum Biochemistry Parameters

After the thawing, the serum samples were used to detect total cholesterol (TC), total triglyceride (TG), total protein (TP), glucose (GLU) and albumin (ALB) contents, as well as the activities of alanine aminotransferase (ALT), aspartate aminotransferase (AST), acid phosphatase (ACP), alkaline phosphatase (AKP) and lysozyme (LZM) as per the manufacturer’s protocol of commercial kits (Jian Cheng Biochemical Co., Ltd., Nanjing, China). Moreover, the serum diamine oxidase (DAO), D-lactate (D-Lac) and lipopolysaccharide (LPS), were used to analyze intestinal permeabilities. Specifically, the activity of DAO and the level of D-Lac were measured by ELISA kits following manufacturer’s protocol (Jian Cheng Biochemical Co., Ltd., Nanjing, China). The value of LPS was detected with a fish specific ELISA kit (Shanghai Enzyme-linked Biotechnology Co., Ltd., Shanghai, China).

### 2.8. Determination of Antioxidant Enzyme Activities in the Liver

In order to assess the liver antioxidant capacity, the frozen pooled tissues were first thawed and homogenized in ten volumes of ice-cold PBS. After centrifugation at 10,000× *g* for 10 min at 4 °C, the resulting supernatants were collected and used for antioxidant measurement. Activities of total superoxide dismutase (T-SOD), catalase (CAT) and glutathione peroxidase (GSH-Px), as well as concentration of malondialdehyde (MDA), were measure by the commercially available reagent kits (Jian Cheng Biochemical Co., Ltd., Nanjing, China).

### 2.9. Intestinal Digestive Enzymes

To elevate the impact of ETSP on intestinal digestive enzyme activity, largemouth bass were fasted for 24 h and the whole intestines were collected, weighted and homogenized in 0.01 M Tris buffer at 1:9 (tissue:buffer) ratio. The supernatants were collected after centrifugation at 10,000× *g* for 10 min at 4 °C and used to detect the activities of total protease, trypsin, α-amylase and lipase, respectively, with the commercially available kits (Jian Cheng Biochemical Co., Ltd., Nanjing, China) following the manufacturer’s instructions.

### 2.10. Intestinal Morphological Analysis

The hematoxylin-eosin (H&E) and Alcian blue-periodic acid-Schiff (AB-PAS) staining was used to estimate the effect of ETSP on intestine morphology and mucus production related parameters, as previously reported [19]. Standard histological techniques were applied to process the fixed mid-intestine and pyloric caeca. The tissues were routinely dehydrated in ascending ethanol concentrations, cleared with xylene, and embedded in paraffin wax. Thereafter, 5 μm-thick sections were prepared and mounted onto pre-treated slides, followed by rehydration and separate H&E and AB-PAS staining. Finally, histopathological images were obtained using an imaging microscope (BX60, Olympus, Tokyo, Japan) equipped with an image acquisition software (CellSens standard, Olympus, Tokyo, Japan).

### 2.11. Total RNA Extraction and Quantitative Real-Time PCR

Total RNA of intestine was extracted by RNAiso Plus Kit (TaKaRa, Beijing, China). Its integrity and concentration were determined by 1% denaturing agarose electrophoresis and measured by Nanodrop 8000 spectrophotometer (ThermoFisher Scientific, Waltham, MA, USA), respectively. After that, 1000 ng of total RNA from each sample was reverse-transcribed into complementary DNA (cDNA) using a PrimeScript^TM^ RT reagent kit with gDNA Eraser (TaKaRa, Dalian, China). Subsequently, the resulting cDNA was diluted to a final concentration of 100 ng/μL with nuclease-free water, and subjected to RT-qPCR using ABI 7500 real-time PCR system (Applied Biosystems, Foster City, CA, USA) with SYBR^®^ Premix ExTaq™ II Kit (Takara, Dalian, China). qRT-PCR was conducted to quantify the transcription profiles of the genes encoding chemokine (*IL-8)*, anti-inflammatory cytokine (*IL-10*), pro-inflammatory cytokines (*IL-1β* and *TNFα*), amino acid transports (*LAT1* and *LAT2*), peptide transports (*PEPT1* and *PEPT2*) and tight junction components (*Occludin*, *Claudin*, *ZO-1* and *ZO-2*). The thermal profile was: 95 °C for 10 min for activation, followed by 40 cycles at 95 °C for 15 s, 60 °C for 1 min, and 72 °C for 30 s. To standardize the gene expression, *β-actin* was used as the housekeeping gene. The relative expression of each target gene was calculated using the 2^−ΔΔCT^ method. The specific primers used in this study are listed in Table 2 and synthesized by Tsingke (Wuhan, China).

### 2.12. Statistical Analysis

The experimental data were expressed as mean ± standard deviation (mean ± SD). All data were analyzed by One-way analysis of variance (ANOVA) using Statistical Package for the Social Sciences (SPSS) version 24.0 software (IBM, Armonk, NY, USA) followed by Tukey’s multiple range test for post hoc comparisons. Differences were considered statistically significant when *p* < 0.05. GraphPad prism 5.0 (GraphPad Software Inc., La Jolla, CA, USA) was used to plot figures.

## 3. Results

### 3.1. Growth Performance and Feed Utilization

Effects of replacing FM with graded ETSP levels on growth performance and feed utilization of largemouth bass were present in Table 3. Initial body weight (IBW) did not differ significantly among groups at the start of the trial (*p* > 0.05). Following the 8-week feeding trial, all fish grew two or three times of their IBW. The values of FBW, WG and SGR were significantly (*p* < 0.05) increased in both H2-25.0 and H2-37.5 groups, in comparison with other groups. Whereas, the FCR in H2-25.0 group was remarkably (*p* < 0.05) lower than other groups. Fish fed ETSP-supplemented diets had lower VSI, LSI, SSI and IFR while higher CF than that of fish fed the control diet. Moreover, there was no significant difference on SR and ISI among all groups. Interestingly, the FI indices in both H2-37.5 and H2-50.0 groups were significantly (*p* < 0.05) high than other groups.

### 3.2. Body Composition of Whole Fish

As shown in Table 4, dietary ETSP supplementation had no significant effect on whole-body composition of largemouth bass, including moisture, crude protein, ash, and crude lipid contents, across all groups (*p* > 0.05). Specifically, the moisture contents were in the range of 72.35 ± 0.43 to 73.92 ± 0.83, the crude protein contents were in the range of 15.74 ± 1.31 to 16.99 ± 0.34. Those for crude lipid contents were 7.10 ± 0.41 to 7.63 ± 0.31, and those for ash contents were 3.48 ± 0.17 to 3.71 ± 0.29.

### 3.3. Effects of Dietary ETSP on Liver Antioxidant Parameters of Largemouth Bass

The potential effect of graded levels of FM replacement by ETSP on antioxidant parameters were measured using hepatic T-SOD, CAT and GSH-Px activities along with the MDA level as indicators. Table 5 showed the antioxidant activity of largemouth bass fed with control or ETSP supplemented diets for eight weeks. T-SOD and GSH-Px activities reached the highest level at H2-50.0 group, while the consent of MDA reached the lowest in H2-50.0 group. Meanwhile, replacing FM with ETSP resulted in a significant increase in CAT activity both in H2-12.5 and H2-50.0 groups (*p* < 0.05).

### 3.4. Effects of Dietary ETSP on Hematological Parameters and Immune Enzyme Activities of Largemouth Bass

The impact of replacing FM with graded levels of ETSP on serum biochemical indices and immune enzyme activities of largemouth bass was described in Table 6. The content of TP in H2-12.5, H2-25.0 and H2-37.5 groups upregulated remarkably (*p* < 0.05) in comparison to control and H2-50.0 groups. The value of ALB was increased proportionally as ETSP supplement increased, reached the highest level in H2-37.5 group and then decreased. In addition, the activities of AST in H2-25.0, H2-37.5 and H2-50.0 groups were significantly lower than other groups. However, graded ETSP supplementation had no significant effect (*p* > 0.05) on serum TG, GLU, TC and ALT among all treatments. The potential effect of dietary ETSP on immune status was analyzed by detecting serum activities of LZM, ACP, and AKP. With the increase in ETSP replacement level, the LZM and ACP activities increased and reached the maximum value in H2-37.5 group (*p* < 0.05). Meanwhile, the activity of AKP in the H2-37.5 group was significantly (*p* < 0.05) higher than that of the Control, H2-25.0 and H2-50.0 group.

### 3.5. Effects of Dietary ETSP on Digestive Enzyme Activities of Largemouth Bass

As shown in Table 7, replacing FM with graded levels of ETSP exerted varying effects on digestive enzyme activities. The total protease activity was significantly (*p* < 0.05) decreased with ETSP levels increasing, and reached the lowest value in H2-50.0 group. Compare with other groups, the trypsin activity in the intestine was significantly lower (*p* < 0.05) in fish fed the H2-25.0 diet. In addition, α-Amylase activity rose remarkably (*p* < 0.05) with increasing FM replacement by ETSP, and peaking in the H2-50.0 group. Whereas, no significant difference (*p* > 0.05) in lipase activity were detected among all the groups.

### 3.6. Effects of Dietary ETSP on Mid-Intestine Morphology of Largemouth Bass

**Figure 1:**
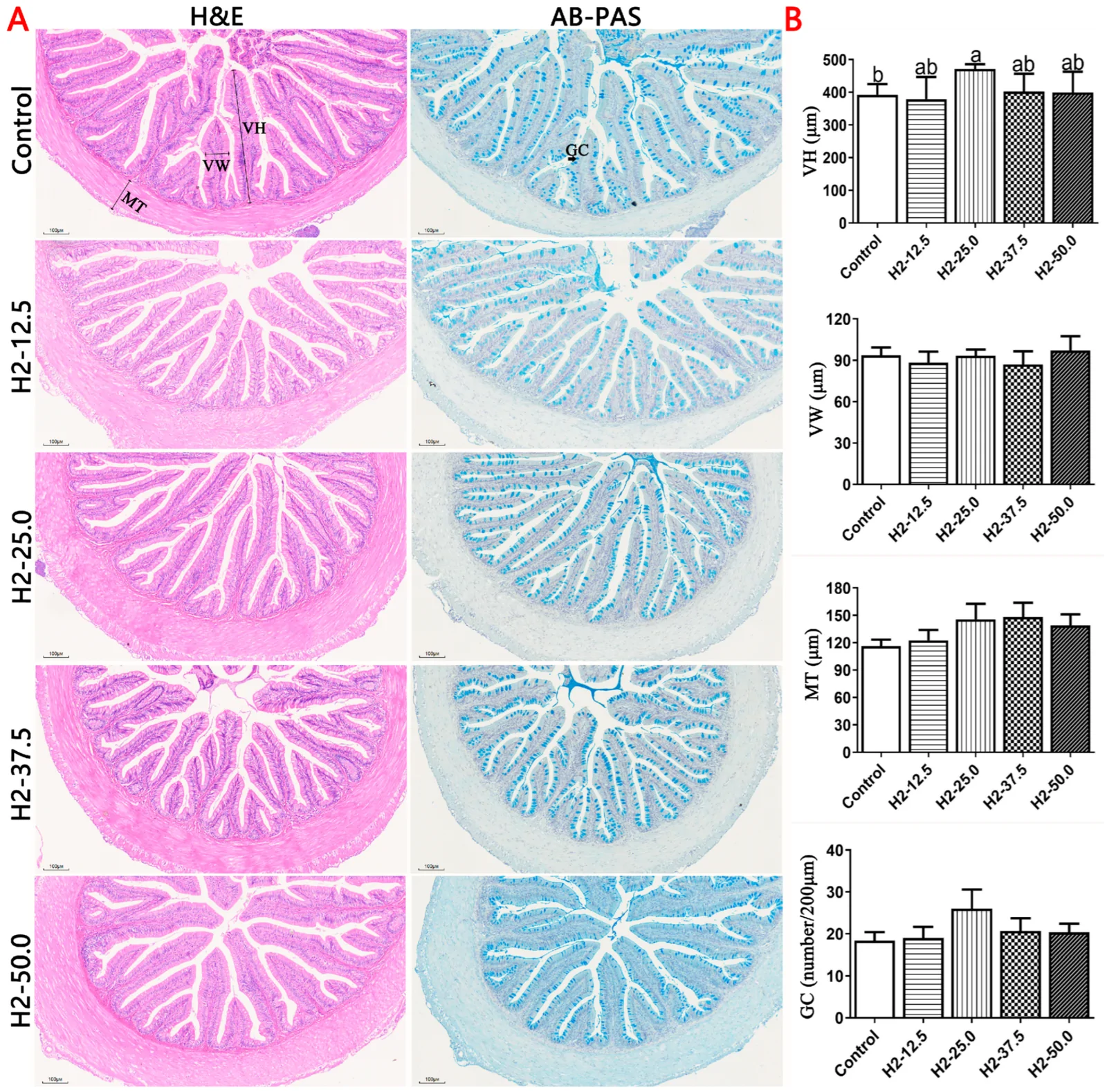
Effects of ETSP on mid-intestine morphology of largemouth bass after the 8-week feeding trial. (**A**) H&E and AB-PAS staining, scale bar = 100 μm; (**B**) Statistical results of VH: villus height; VW: villus width; MT: muscle thickness and, GC: goblet cells. All values are expressed as the means ± SD. Different superscript letters indicate significant difference among groups (*p* < 0.05).

After 8 weeks’ feeding trial, effects of graded levels of FM replacement by ETSP on morphology index in the mid-gut of largemouth bass were shown in Figure 1, and the structure of each layer of intestinal tissue at ETSP-treated fish was clear. Abundant and regularly arranged intestinal villi were observed, with intact mucosal epithelial cells and numerous goblet cells showing no obvious abnormalities. Among all replacement levels of FM with ETSP, the VW and MT values as well as the GC counts were not significantly affected (*p* > 0.05). Interestingly, the MT values and GC number showed a tendency of increased firstly, reached the highest level at H2-25.0 group, and then decreased at H2-50.0 group. Importantly, the VH value was significantly (*p* < 0.05) increased in H2-25.0 group in comparison with the control group.

### 3.7. Effects of Dietary ETSP on Pyloric Caeca Morphology of Largemouth Bass

**Figure 2:**
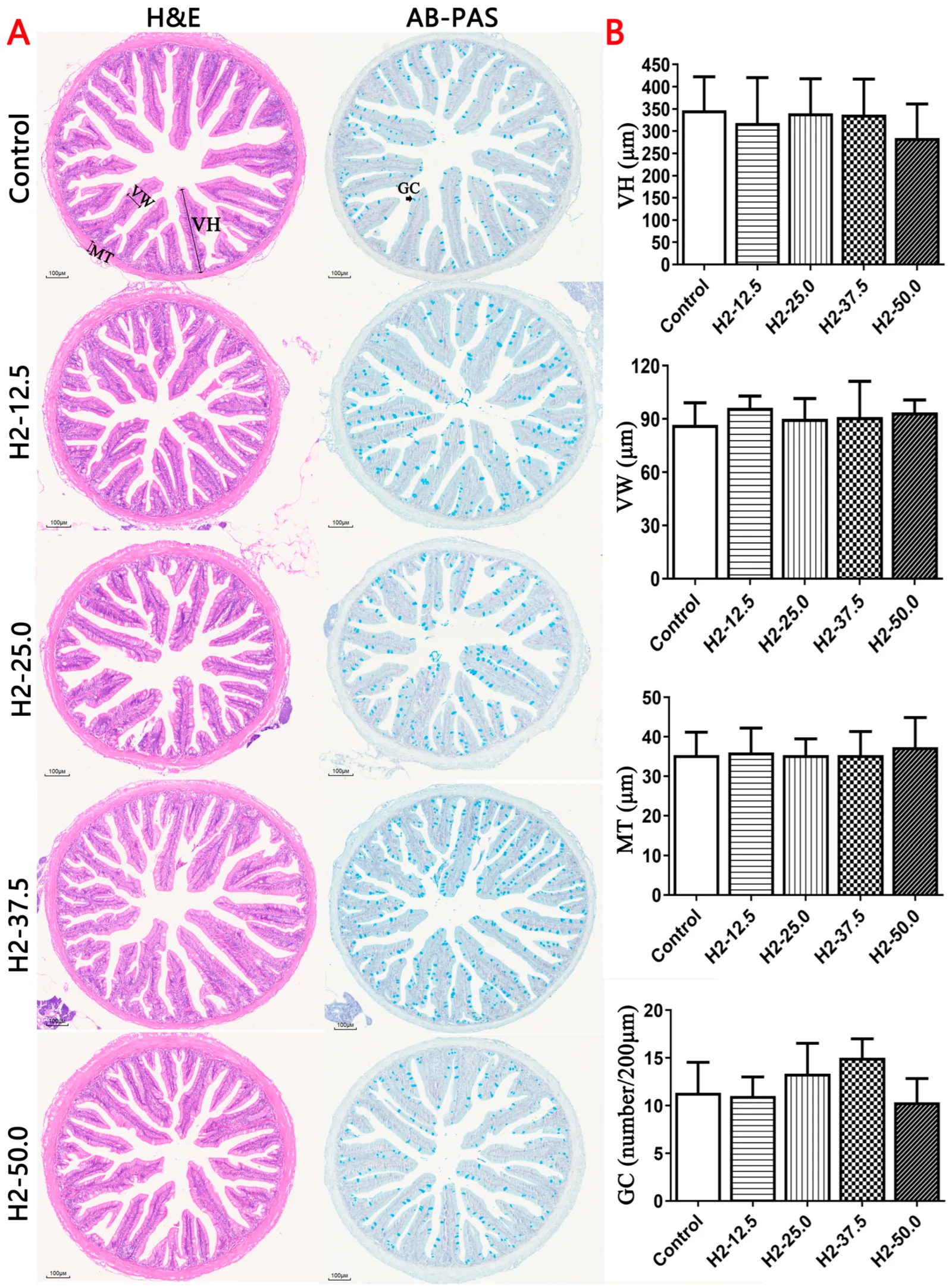
Effects of ETSP on pyloric caeca morphology of largemouth bass after the 8-week feeding trial. (**A**) H&E and AB-PAS staining, scale bar = 100 μm; (**B**) Statistical results of VH: villus height; VW: villus width; MT: muscle thickness and, GC: goblet cells. All values are expressed as the means ± SD.

H&E and AB-PAS staining were used to examine the pyloric caeca morphology of largemouth bass fed experimental diets. An intact epithelial barrier with abundant mucosal folds and numerous goblet cells was observed across all dietary treatment groups (Figure 2). The amount of GC showed a tendency of upregulated firstly, reached the maximum level at H2-37.5 group, and then decreased at H2-50.0 group. Moreover, dietary ETSP has no significant effects on VH, VW and MT values of largemouth bass pyloric caeca in comparison with the control group (*p* > 0.05).

### 3.8. Effects of Dietary ETSP on Intestinal Permeability

**Figure 3:**
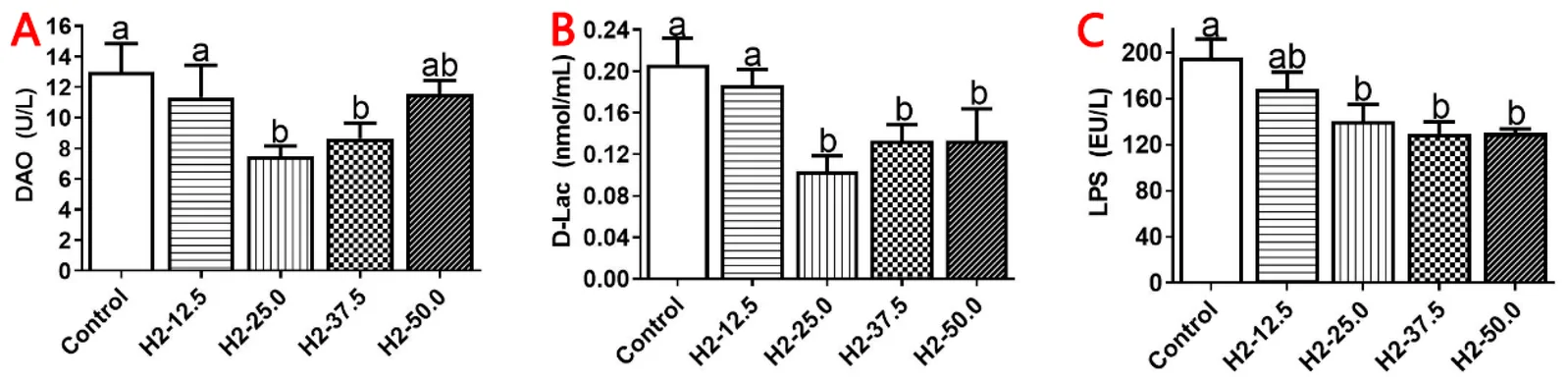
Intestinal permeability parameters of largemouth bass fed diets with ETSP for replacement of fish meal after 8 weeks. (**A**) DAO: diamine oxidase; (**B**) D-Lac: D-lactate; (**C**) LPS: lipopolysaccharide. Values are expressed as means ± SD (n = 6). Results with significant differences were marked with different letters (*p* < 0.05).

**Figure 4:**
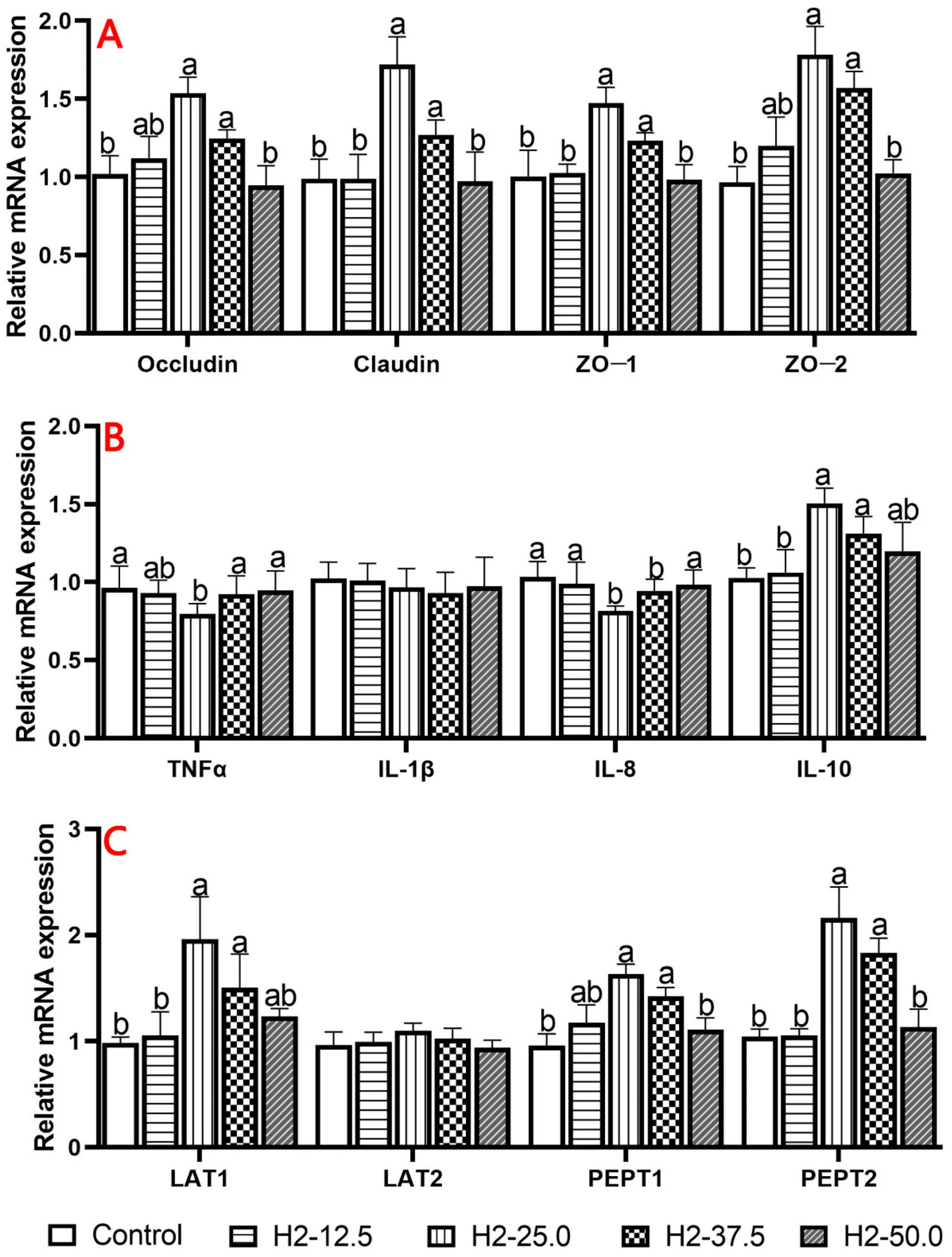
The effects of replacing FM with ETSP in feed on gene expression levels in intestine of largemouth bass after the 8-week feeding trial. (**A**) Relative transcription levels of tight junction genes; (**B**) Relative transcription levels of inflammatory cytokines genes; (**C**) Relative transcription levels of peptide and amino acid transporter. Values are expressed as means ± SD (n = 6). Results with significant differences were marked with different letters (*p* < 0.05).

To explore whether graded levels of FM replacement by ETSP affects the variations in the intestinal permeability, we analyzed the activity of DAO and the concentration of D-Lac and LPS in serum as well as the expression of tight junction related genes in intestine. Specifically, the DAO activity and D-Lac level show a tendency of significantly (*p* < 0.05) decreased with ETSP levels increasing, reached the lowest level in H2-25.0 group, and then increased (Figure 3A,B). Meanwhile, the lowest level of LPS in serum was detected in the H2-50.0 group (*p* < 0.05), with no significantly different from those in H2-25.0 and H2-37.5 groups (Figure 3C). The transcription level of *Occludin*, *Claudin*, *ZO-1* and *ZO-2* were significantly (*p* < 0.05) increased with ETSP levels increasing, reached the highest level in H2-25.0 group, and then decreased with future increasing substitution levels (Figure 4A).

### 3.9. Effects of Dietary ETSP on Genes Related to the Inflammatory Response

To explore whether graded levels of FM replacement by ETSP affects the variations in the intestinal inflammatory response, the relative transcription levels of inflammatory cytokines genes were analyzed. The results were shown in Figure 4B, the transcription levels of *IL-8* and *TNFα* gene were both significantly (*p* < 0.05) decreased with ETSP levels increasing, reached the minimum level in H2-25.0 group, and then increased. Conversely, the expression of *IL-10* showed an opposite trend. Whereas, no obvious difference was observed in *IL-1β* mRNA expression with the increasing dietary ETSP inclusion levels (*p* > 0.05).

### 3.10. Effects of Dietary ETSP on Expression of Genes Related to Amino Acid and Oligopeptide Transporter

The effects of replacing FM with graded levels of ETSP on the expression of genes associated with amino acid and oligopeptide transporters were measured and displayed in Figure 4C. All transcription levels of *LAT1*, *PEPT1* and *PEPT2* exhibited a similar tendency to upregulate first, reach the peak values in H2-25.0 group, and then downregulate. Specifically, the *LAT1* and *PEPT2* mRNA levels in fish intestine revealed no apparent (*p* > 0.05) difference among H2-12.5, H2-50.0 and control groups. Nevertheless, the expression of those genes in H2-25.0 and H2-37.5 groups upregulated significantly (*p* < 0.05) than other treatments. For mRNA expression of *PEPT1*, significant (*p* < 0.05) increase was observed in H2-25.0 and H2-37.5 groups compared other groups, while there was no statistically significant (*p* > 0.05) difference among H2-12.5, H2-50.0 and control groups in case of this gene expression. Whereas, the transcription level of *LAT2* was not significantly affected by replacing FM with ETSP.

## 4. Discussion

Growth performance is a particularly important characteristic in aquaculture, as it is directly correlated with both productivity and profitability. Therefore, growth performance is the often considered as a key indicator to analyze aquafeed quality [20]. Previous study was carried out to explore the potential of replacement of FM with ETSP on growth performance and feed efficiency in Gibel carp (*Carassius auratus gibelio*), which were fed with diets containing different levels of ETSP (0%, 4%, 8%, 12%, 16%, and 20%) for 159 days. The results indicated that growth performance and whole-body composition did not differ significantly between the control group and the other groups receiving various replacement levels [21]. In another research, juvenile hybrid grouper (*Epinephelus fuscoguttatus ♀* × *E. lanceolatus ♂*) were fed for 8 weeks with four experimental diets supplemented with soybean protein hydrolysates at levels of 0, 10, 20, 40 and 60 g/kg. The results revealed that the FW, FCR, SGR and WG were not significantly affected when complete replacement of FM with ETSP [22]. In this study, with ETSP levels increasing the FBW, WG and SGR were initially increased and then decreased, reaching the maximum value in H2-25.0 group, implying that replace FM with ETSP have certain effect on fish growth. Consistent results were reported by Liu et al. [23], who investigated the effects of substitution FM with ETSP in the feed of juvenile largemouth bass (*M. salmoides*). These findings suggest that ETSP sever as a promising alternative for FM in the largemouth bass diet. The replaceability of ETSP in aquafeeds is attributed to the increased levels of small peptides and free amino acids as well as reduced antinutritional factors when soybean is treated with specific enzymes.

The indicators, such as CF, HSI and VSI, partially represent the body lipid, lean, and growth of aquatic animals [24]. Moreover, the nutritional condition in fish can be detected using the HSI, which indirectly indicates hepatic glycogen levels. Lower values of HSI may be indicative of stress. Both HSI and VSI are utilized to assess whether energy is being reallocated from organ or tissue growth to cope with stress [25]. In the present research, no remarkable changes in the HSI and VSI were detected across all replacement levels, indicating that substituting FM with ETSP, even reaching 50%, did not disturb glycogen and carbohydrate energy reserves. Similar results were also reported by Liu et al. [23] in juvenile largemouth bass fed diets with ETSP replacing FM.

Nutrient digestion and absorption are closely related to individual growth [26]. Proteins are absorbed by animals in the form of amino acids and small peptides, which are translocated across membranes by specific transporters [27]. It has been shown in previous work that enzyme-treated soybean can be easily absorbed to further develop their physiological function, primarily because of their high contents of amino acids and small peptides [27]. Hence, the variations in growth performance observed in fish may be attributed to differential absorption of amino acids and small peptides. LAT1 and LAT2 are both L-type transporters which act a key role in transporting neutral amino acids [28]. Whereas, LAT1 mainly involving transportation of large neutral amino acids with branched or aromatic side chains, and LAT2 exhibit broad specificity for both small and large zwitterionic amino acids [29]. Previous studies have found that substitution FM with soybean protein concentrate hydrolysate increased transcription levels of *LAT1* and *LAT2* in largemouth bass [30] and mandarin fish (*Siniperca chuatsi*) [31]. Similarly, this study found that the transcription level of *LAT1* was quadratically increased in response to dietary ETSP supplementation, and the H2-25.0 group reached the maximum value, implying that proper amount of ETSP enhances the amino acid absorption capacity of largemouth bass. PEPT1 and PEPT2 are responsible for the translocation of dipeptides and tripeptides released from large protein [32]. In this research, proper amount of ETSP instead of FM can upregulate intestinal *PEPT1* and *PEPT2* genes expression, possibly due to the high content of tripeptides and dipeptides in ETSP. It is noteworthy that the transcription value of those genes gradually downregulated when the amount of ETSP was further increased. This might be due to the fact that ETSP have abundant free amino acids, which can saturate the intestinal transport system and cause transport competition, thereby suppressing the expression of genes associated with amino acid transport [33]. Similar results were observed on yellow perch (*Perca flavescens*) [34] and largemouth bass [35]. Therefore, we can conclude that partial replacement of FM with ETSP promotes growth performance of host is associated with the improvement of amino acid and small peptides through increasing *LAT1*, *PEPT1* and *PEPT2* expression levels.

The hematological parameters are commonly use as indicators to directly and indirectly reflect the general health status, physiological stress level and nutrient metabolism of aquatic animals fed with different experimental diets [36]. The levels of serum TP and ALB are closely associated with the state of fish health and protein metabolism, and upregulation of those parameters reflects enhanced absorption and metabolic activity, which in turn increases protein synthesis and nitrogen deposition [37]. In this research, supplementation of ETSP to replace FM significantly increased contents of serum TP and ALB, which aligns with previous findings in Gibel carp (*C. auratus gibelio*) fed with a diet inclusion of ETSP for 159 days [21]. Conversely, Liang et al. reported that substituting FM with soybean protein hydrolysates in the diet of juvenile hybrid grouper (*E. fuscoguttatus ♀* × *E. lanceolatus ♂*) remarkably decreased the serum TP and had no effects on serum ALB [22]. The levels of TC and TG in serum are regarded as indicators of health status, lipid metabolism and immune function in aquatic animals [38]. In addition, the serum GLU is important biomarker of stress, and its value is associated with energy induced by a stressor [39]. In the present research, the levels of TC, TG and GLU did not change significantly at any replacement level, suggesting that replacing FM with ETSP does not significantly induce stress and impar fish health. AST and ALT, two important aminotransferases predominantly distributed in fish liver, will release into the blood when liver injury occurs [40]. Therefore, the AST and ALT activities are often utilized to assess the physiological state of the liver in fish [41]. In the present research, the AST revealed lower values in the ETSP substitution groups, while the ALT was no significantly affected, implying that the liver physiology was not impaired by replacing FM with ETSP. Similar results are also found in Gibel carp (*C. auratus gibelio*) [21].

The digestive enzymes including protease, lipase and amylase were proved to be affected by feeding behavior, dietary composition and nutrient levels [42]. Therefore, activities of these enzymes partly reflect fish digestive capacity to some extent, and measuring them is crucial for evaluating the nutritional physiology and feed utilization of aquatic animals [43]. This research showed that α-amylase activities in intestine increased with substantial of ETSP, suggesting that replacing FM with ETSP improves intestinal amylase activity, thereby providing nutrients to support fish growth. The finding agrees well with previous results that replacing FM with ETSP in the diets of European sea bass (*Dicentrarchus labrax*) have a significant stimulation effect on amylase enzymes [44]. Whereas, the activities of total protease and trypsin were decreased in ETSP substitution group, the reason was perhaps due to fact that the high molecular protein was hydrolyzed into small peptides and amino acids.

For aquatic animals, a proper balance between production and scavenging of reactive oxygen species (ROS) is crucial for preserving normal metabolic homeostasis [45]. The produced ROS was catalyzed into H_2_O_2_ and O^2−^ by SOD and then converted into H_2_O and O_2_ through GPx or CAT [46]. Thus, measuring these enzyme activities is often utilized to evaluate antioxidant function and served as a biomarker of oxidative stress status in animals. Malondialdehyde (MDA) level, which represents the degree of phospholipid peroxidation in tissues, is widely regarded as an indicator for evaluating the degree of lipid peroxidation in aquatic organisms [47]. The result demonstrates that replacing FM with ETSP at certain level improve liver T-SOD, CAT and GSH-Px activities while reducing MDA content. The finding is concurred with previous studies on largemouth bass [48] and Jian carp (*Cyprinus carpio* var. Jian) [49] when substituting FM with ETSP, implying that applying ETSP as alternatives to FM eliminated peroxidation products and improved antioxidative activity.

The intestinal tract is the main organ for digestion and absorption, and its morphological changes are strongly correlated with digestive and absorptive functions [50]. Therefore, intestinal morphology is frequently used to assess the impact of diets on growth performance of aquatic animals [51]. Micromorphological features, including VH and VW values as well as goblet cell numbers, are important parameters that can represent the intestinal digestive and absorptive function [52]. Generally, increasing the surface area available for digestion and absorption helps intestinal mucosal epithelial cells more efficiently digest chyme and absorb nutrients into the bloodstream [53]. Mucus secreted by goblet cells exert bactericidal effects by capturing pathogens and limiting their adherence to the intestinal mucosa [54]. The present study showed that juvenile fish in the H2-25.0 group had a higher VH value than those in other groups. This finding aligns with the observation that adding enzymatically hydrolyzed compound soy protein to the diet significantly increase the intestinal VH value of American eels (*Anguilla rostrata*) [55]. Therefore, increasing VH values improves digestive and absorptive capacity of juvenile fish, which in turn promotes their growth.

The integrity of intestinal physical barrier is fundamental to fish intestinal immunity and is crucial for resisting enteric pathogen invasion [56]. DAO is an intracellular enzyme mainly located in the intestinal epithelial cell [57], D-Lac is a bacterial metabolite usually found in the intestinal mucosa [58], and LPS is a component of the cell wall of Gram-negative bacteria [59]. An intact intestinal mucosal barrier prevents these three substances translocating from the epithelium into blood [60]. It has been frequently reported that raised permeability and ruined integrity of intestine are correlated with increased serum DAO activity as well as D-Lac and LPS concentration [61]. In the present study, the activity of DAO and the contents of D-Lac and LPS in serum were gradually decreased when replacing FM with ETSP in the diets.

It is well known that the integrity of intestinal physical barrier mainly determined by tight junction, and the pathogen will invade host from the intestine when the tight junction was damaged [62]. Occludin, claudins, and other transmembrane proteins, together with intracellular plaque proteins like ZO-1 and ZO-2, constitute tight junctions [63]. In our research, partial replacing FM with ETSP obviously increased these gene expression in the intestine. This finding is consistent with results obtained when replacing FM with ETSP in the diet of largemouth bass [48]. The decreased activity of DAO and the concentration of D-Lac and LPS in serum coincided with upregulation of intestinal transcription value of *occludin*, *claudins*, *ZO-1* and *ZO-2* genes suggested that appropriate ETSP alternative to FM improved intestinal physical barrier function. Moreover, the increased integrity of intestinal physical barrier can also be reflected by inhibition of inflammatory response by decreasing *IL-8* and *TNFα* as well as increasing *IL-10* expression levels.

## 5. Conclusions

In summary, at the fishmeal level used in the diet tested in the present study, 25% replacement is the most beneficial for juvenile largemouth bass as determined by WG, SGR and FCR, respectively. The outcomes of the present research indicated that replacement of FM with ETSP at an appropriate dose has beneficial effects on the growth performance, antioxidant status, intestinal barrier function and nutrient transport, suggesting that ETSP is a promising candidate to replace FM in juvenile largemouth bass diets. In future, further studies could be focused on the bioactive substances in ETSP on the immune response of fish and the specific mechanism of ETSP in modulating fish intestine structure.

## Figures and Tables

**Table 1:** Formulation and proximate composition of the experimental diets.

Ingredient (%)	Control	H2-12.5	H2-25.0	H2-37.5	H2-50.0
Fish meal ^a^	400.0	350.0	300.0	250.0	200.0
Enzyme-treated soybean meal ^b^	0.0	57.7	115.4	173.1	230.9
Soybean meal	100.0	100.0	100.0	100.0	100.0
Corn gluten meal	100.0	100.0	100.0	100.0	100.0
Chicken meal	120.0	120.0	120.0	120.0	120.0
Wheat flour	140.0	140.0	140.0	140.0	140.0
Microcrystalline cellulose	58.1	45.3	32.8	20.1	7.4
Fish oil	10.0	10.0	10.0	10.0	10.0
Soybean oil	5.9	10.0	13.8	17.8	21.7
Lecithin oil	20.0	20.0	20.0	20.0	20.0
Mineral premix ^c^	10.0	10.0	10.0	10.0	10.0
Vitamin premix ^d^	10.0	10.0	10.0	10.0	10.0
Choline chloride	5.0	5.0	5.0	5.0	5.0
Calcium biphosphate	10.0	10.0	10.0	10.0	10.0
Lysine	5.0	5.5	6.0	6.5	7.0
Methionine	5.0	5.5	6.0	6.5	7.0
Yttrium oxide	1.0	1.0	1.0	1.0	1.0
Total	1000.0	1000.0	1000.0	1000.0	1000.0
Moisture	7.83	7.16	7.69	7.89	7.73
Crude protein (% DM)	46.57	46.58	46.46	45.64	46.59
Ash (% DM)	7.51	7.63	7.72	7.31	7.27
Crude lipid (% DM)	10.07	10.11	10.13	10.09	10.09
Ca	1.07	1.13	1.09	1.04	1.02
P	0.93	0.97	0.92	0.94	1.00

Note: Enzyme-treated soybean protein (ETSP) was provided by China National Food and Oil Corporation (Beijing, China), and other ingredients were supplied by Dachang Biotechnology Co., Ltd., Fujian, China. ^a^ Fish meal: 65.85% of crude protein and 11.39% of crude lipid. ^b^ Enzyme-treated soybean meal (ETSP): 57.05% of crude protein and 3.09% of crude lipid. ^c^ Mineral premix, mg/kg diet: NaCl, 100; MgSO_4_·7H_2_O, 2000; NaH_2_PO_4_·2H_2_O, 3300; KH_2_PO_4_, 4300; Fe-citrate, 300; ZnSO_4_·7H_2_O, 141.2; CoCl_2_·6H_2_O, 0.4; KIO_3_, 1.2; MnSO_4_·H_2_O, 64.8; CuSO_4_·5H_2_O, 12.4; Na_2_SeO_3_·5H_2_O, 0.61. ^d^ Vitamin premix, mg/kg diet: Thiamine, 120; Riboflavin, 100; Folic acid, 30; Nicotinic acid, 800; Vitamin B6, 40; Calcium pantothenate, 200; Inositol, 4000; Biotin, 12; Vitamin B12, 0.18; Vitamin A, 100,000 IU; Vitamin D, 2000 IU; Vitamin E, 450; Vitamin K3, 80.

**Table 2:** Genes and primer sequences used for qRT-PCR.

Target Genes	F: Primer Sequence (5′-3′)	Amplicon Size (bp)	Accession No.
*TNFα*	F: CTTCGTCTACAGCCAGGCATCG	161	XM_038710731.1
	R: TTTGGCACACCGACCTCACC		
*IL-1β*	F: CGTGACTGACAGCAAAAAGAGG	166	XM_046034892.1
	R: GATGCCCAGAGCCACAGTTC		
*IL-8*	F: CGTTGAACAGACTGGGAGAGATG	107	MW751832.1
	R: AGTGGGATGGCTTCATTATCTTGT		
*IL-10*	F: AGCCAGCAGCATCATTACCA	118	XM_038696252.1
	R: AGAACCAGGACGGACAGGAG		
*LAT1*	F: CCAAAGCACGAC AGACCTACA	136	XM_038706332.1
	R: ACCAACCTGGCATATT TCACC		
*LAT2*	F: GGTGACCACAGG GATAGAGATG	104	XM_038718738.1
	R: TTGCTTACGGAGGCTG GAACTT		
*PEPT1*	F: AGAAAGGGAAGAAGAGCAGAG	137	MZ773078.1
	R: CTTAAAGTACAGGACCAGGACA		
*PEPT2*	F: CAAGTCAGTTGGAGCCATTCC	249	XM_038721077.1
	R: TGCACATCCCCTCTCAGTACG		
*Occludin*	F: GATATGGTGGCAGCTACGGT	198	XM_038715419.1
	R: TCCTACTGCGGACAGTGTTG		
*Claudin*	F: TGCTGGTCTGCTTTCTCTGGT	212	XM_038718401.1
	R: GTGGGTAGCGTGGAGGTTTC		
*ZO* *-* *1*	F: ATCTCAGCAGGGATTCGACG	208	XM_038701018.1
	R: CTTTTGCGGTGGCGTTGG		
*ZO* *-* *2*	F: TGGCAGCATTCGGATCGATTA	235	XM_038702595.1
	R: ACATCAGCAGAGTCCATTAGCAC		
*β-actin*	F: TTCACCACCACAGCCGAAAG	179	MH018565.1
	R: TCTGGGCAACGGAACCTCT		

Notes: *TNFα:* tumor necrosis factor α, *IL-1β*: interleukin 1β, *IL-8*: interleukin 8, *IL-10*: interleukin 10, *LAT1*: L-type amino acid transporter 1, *LAT2*: L-type amino acid transporter 2, *PEPT1*: oligopeptide transporter 1, *PEPT2*: oligopeptide transporter 2, *ZO-1*: zona occluding-1, *ZO-2:* zona occluding-2.

**Table 3:** The growth, feed utilization and physical indices of largemouth bass fed diets with graded levels of ETSP (g/kg diets) for 8 weeks.

Parameters	Control	H2-12.5	H2-25.0	H2-37.5	H2-50.0
SR (%)	100 ± 0.00	98.89 ± 1.92	98.89 ± 1.92	98.89 ± 1.92	100 ± 0.00
IBW (g/fish)	15.55 ± 0.01	15.43 ± 0.05	15.49 ± 0.10	15.48 ± 0.04	15.44 ± 0.10
FBW (g/fish)	53.27 ± 0.73 ^b^	52.13 ± 2.96 ^b^	60.04 ± 4.78 ^a^	59.41 ± 1.26 ^a^	53.95 ± 5.27 ^b^
WG (g)	37.72 ± 0.73 ^b^	36.70 ± 2.92 ^b^	44.56 ± 4.70 ^a^	43.93 ± 1.25 ^a^	38.51 ± 5.18 ^b^
SGR (%/day)	2.20 ± 0.02 ^b^	2.17 ± 0.09 ^b^	2.42 ± 0.13 ^a^	2.40 ± 0.04 ^a^	2.23 ± 0.16 ^b^
FI (g/fish)	53.38 ± 2.42 ^b^	50.59 ± 3.45 ^b^	54.80 ± 2.45 ^b^	59.55 ± 1.21 ^a^	58.38 ± 3.01 ^a^
FCR	1.41 ± 0.04 ^ab^	1.38 ± 0.02 ^ab^	1.24 ± 0.09 ^b^	1.36 ± 0.01 ^ab^	1.53 ± 0.15 ^a^
CF (g/cm^3^)	1.23 ± 0.07	1.38 ± 0.15	1.29 ± 0.08	1.35 ± 0.07	1.38 ± 0.09
VSI (%)	9.53 ± 0.62	8.35 ± 1.21	8.09 ± 1.02	8.01 ± 1.11	8.90 ± 0.94
LSI (%)	3.71 ± 0.73	2.89 ± 0.85	2.32 ± 0.69	2.20 ± 0.54	2.99 ± 0.82
ISI (%)	1.96 ± 0.22	1.98 ± 0.39	1.92 ± 0.18	1.88 ± 0.30	1.99 ± 0.14
SSI (%)	1.17 ± 0.18	1.04 ± 0.12	1.05 ± 0.20	1.17 ± 0.11	1.09 ± 0.13
IFR (%)	2.42 ± 0.46	1.70 ± 0.49	1.79 ± 0.69	2.05 ± 0.67	1.99 ± 0.66

Values in same line with different superscripts shows significant difference (*p* < 0.05). SR: Survival rate, IBW: Initial body weight, FBW: Final body weight, WG: Weight gain, SGR: Specific growth rate, FI: Feed intake, CF: Condition factor, FCR: Feed conversion ratio, VSI: Viscera somatic index, LSI: liver somatic index, ISI: intestine somatic index, SSI: stomach somatic index, IFR: intraperitoneal fat ratio.

**Table 4:** Effects of different levels of dietary ETSP on whole body proximate composition of largemouth bass after 8 weeks.

Parameters	Control	H2-12.5	H2-25.0	H2-37.5	H2-50.0
Moisture	73.92 ± 0.83	73.32 ± 0.38	72.35 ± 0.43	73.42 ± 1.61	73.42 ± 0.92
Protein	15.74 ± 1.31	15.81 ± 0.47	16.99 ± 0.34	16.11 ± 1.27	16.09 ± 0.87
Lipid	7.37 ± 1.54	7.41 ± 0.42	7.10 ± 0.41	7.10 ± 1.33	7.63 ± 0.31
Ash	3.48 ± 0.17	3.50 ± 0.20	3.60 ± 0.48	3.71 ± 0.29	3.55 ± 0.38

**Table 5:** Effects of different levels of dietary ETSP on the liver antioxidant capacity of *M. salmoides*.

Parameters	Control	H2-12.5	H2-25.0	H2-37.5	H2-50.0
T-SOD (U/mg prot)	43.49 ± 1.11 ^c^	45.01 ± 1.38 ^b^	46.11 ± 0.42 ^b^	45.43 ± 2.20 ^b^	50.32 ± 1.94 ^a^
CAT (U/mg prot)	2.56 ± 0.14 ^b^	3.45 ± 0.21 ^a^	2.97 ± 0.11 ^ab^	2.85 ± 0.49 ^b^	3.05 ± 0.45 ^a^
GSH-Px (U/mg prot)	49.59 ± 0.94 ^c^	46.16 ± 1.69 ^c^	49.76 ± 1.54 ^c^	55.45 ± 2.38 ^b^	60.11 ± 1.24 ^a^
MDA (nmol/mg prot)	2.27 ± 0.29 ^a^	1.76 ± 0.18 ^a^	1.47 ± 0.16 ^b^	1.95 ± 0.43 ^a^	1.35 ± 0.07 ^b^

Values in same line with different superscripts shows significant difference (*p* < 0.05). T-SOD: Total superoxide dismutase, CAT: Catalase, GSH-Px: Glutathione peroxidase, MDA: Malondialdehyde.

**Table 6:** Effects of different levels of dietary ETSP on serum biochemical indices and immune enzyme activities of *M. salmoides*.

Parameters	Control	H2-12.5	H2-25.0	H2-37.5	H2-50.0
TP (mg/mL)	18.39 ± 0.97 ^c^	31.52 ± 2.18 ^a^	27.59 ± 2.46 ^a^	30.62 ± 5.03 ^a^	22.44 ± 2.13 ^b^
ALB (mg/mL)	10.29 ± 1.01 ^b^	14.47 ± 0.92 ^a^	14.07 ± 0.72 ^a^	17.35 ± 1.35 ^a^	16.23 ± 0.62 ^a^
TG (mg/mL)	1.03 ± 0.30	1.10 ± 0.27	1.16 ± 0.28	0.89 ± 0.29	0.81 ± 0.26
GLU (mmol/L)	3.57 ± 0.93	3.68 ± 0.41	3.75 ± 0.16	3.98 ± 0.14	3.98 ± 0.19
TC (mmol/L)	8.05 ± 0.61	8.50 ± 0.46	8.08 ± 0.09	8.16 ± 0.19	8.00 ± 0.15
ALT (U/mL)	4.97 ± 0.20	4.94 ± 0.65	4.74 ± 1.04	4.65 ± 0.45	4.90 ± 0.85
AST (U/mL)	1.68 ± 0.44 ^a^	1.41 ± 0.32 ^a^	1.12 ± 0.19 ^b^	1.29 ± 0.50 ^b^	1.04 ± 0.49 ^b^
LZM (μg/mL)	142.00 ± 9.98 ^b^	153.68 ± 3.39 ^ab^	161.63 ± 7.15 ^a^	176.97 ± 11.22 ^a^	165.12 ± 3.99 ^a^
ACP(U/mL)	11.77 ± 1.22 ^c^	11.29 ± 1.06 ^c^	13.69 ± 1.14 ^b^	16.88 ± 0.40 ^a^	14.22 ± 0.89 ^b^
AKP (U/mL)	11.66 ± 0.62 ^b^	12.95 ± 1.01 ^ab^	11.89 ± 1.17 ^b^	14.14 ± 1.00 ^a^	12.15 ± 0.91 ^b^

Values in same line with different superscripts shows significant difference (*p* < 0.05). TP: Total protein, ALB: Albumin, TG: Total triglyceride, GLU: Glucose, TC: Total cholesterol, ALT: Alanine aminotransferase, AST: Aspartate aminotransferase, LZM: Lysozyme, ACP: Acid phosphatase, AKP: Alkaline phosphatase.

**Table 7:** Effects of different levels of dietary ETSP on intestine digestive enzyme activities of *M. salmoides*.

Parameters	Control	H2-12.5	H2-25.0	H2-37.5	H2-50.0
Total Protease (IU/g intestine)	4.17 ± 0.16 ^a^	3.97 ± 0.67 ^b^	3.83 ± 0.94 ^b^	3.10 ± 0.72 ^c^	2.97 ± 0.67 ^c^
Trypsin (IU/g intestine)	0.96 ± 0.17 ^a^	0.94 ± 0.08 ^a^	0.74 ± 0.03 ^b^	0.84 ± 0.16 ^a^	0.86 ± 0.11 ^a^
Lipase (IU/g intestine)	0.93 ± 0.08	0.88 ± 0.02	0.92 ± 0.10	0.91 ± 0.07	0.86 ± 0.06
α-Amylase (IU/g intestine)	0.36 ± 0.03 ^b^	0.39 ± 0.02 ^b^	0.42 ± 0.01 ^ab^	0.46 ± 0.02 ^a^	0.49 ± 0.02 ^a^

Values in same line with different superscripts shows significant difference (*p* < 0.05).

## Data Availability

The raw data supporting the conclusions of this article will be made available by the authors on request.

## Supplementary Materials

The following supporting information can be downloaded at: https://www.mdpi.com/article/10.3390/ani16162589/s1, Table S1: Proximate composition of the enzyme-treated soybean protein (ETSP) used in the dietary treatment formulation for largemouth bass (*Micropterus salmoides*); Table S2: Contents of amino acid (AA) in enzyme-treated soybean protein (ETSP).
