# Supplementary material for: Effects of Replacing Dietary Fishmeal with Enzyme-Treated Soybean Protein on Growth Performance, Serum Biochemical Indices, Antioxidant, Digestive Enzyme Activities, Intestinal Barrier Integrity and Relative mRNA Expression Levels of Juvenile Largemouth Bass (Micropterus salmoides)
Source: Animals (Basel). 2026 Aug 19;16(16):2589. doi: 10.3390/ani16162589 (PMC13508934; doi:10.3390/ani16162589)
Supplement: Supplementary file 1 [file animals-16-02589-s001.zip › animals-4471310-supplementary.pdf]

Supplement **Table S1** Proximate composition of the enzyme-treated soybean protein (ETSP) used in the dietary treatment formulation for largemouth bass (*Micropterus salmoides*).

Composition	Ash (%)	Dry matter (%)	Crude protein (%)	Crude fat (%)	Ca (%)	P (%)	Acid-soluble protein (%)	soluble protein (%)	Glycinin (mg/g)	β-conglycinin (μg/g)
Percentage (%)	6.44	91.91	57.05	3.09	0.35	0.82	12.51	21.07	1.10	1.06

Supplement **Table S2** Contents of amino acid (AA) in enzyme-treated soybean protein (ETSP).

Composition	Non-essential amino acids								Essential amino acids									
	Asp	Tyr	Ser	Glu	Pro	Gly	Ala	Cys	Val	Met	Ile	Leu	Trp	Phe	His	Lys	Arg	Thr
Percentage (%)	6.04	1.69	2.57	9.33	2.66	2.26	2.37	0.63	2.53	0.58	2.50	4.17	0.68	2.65	1.33	3.35	3.55	2.08
